# Identification of SNAT Family Genes Suggests *GhSNAT3D* Functional Reponse to Melatonin Synthesis Under Salinity Stress in Cotton

**DOI:** 10.3389/fmolb.2022.843814

**Published:** 2022-02-10

**Authors:** Yuexin Zhang, Cun Rui, Yapeng Fan, Nan Xu, Hong Zhang, Jing Wang, Liangqing Sun, Maohua Dai, Kesong Ni, Xiugui Chen, Xuke Lu, Delong Wang, Junjuan Wang, Shuai Wang, Lixue Guo, Lanjie Zhao, Xixian Feng, Chao Chen, Wuwei Ye

**Affiliations:** State Key Laboratory of Cotton Biology / Institute of Cotton Research of Chinese Academy of Agricultural Sciences / Zhengzhou Research Base, School of Agricultural Sciences, Zhengzhou University / Key Laboratory for Cotton Genetic Improvement, MOA, Anyang, China

**Keywords:** serotonin N-acetyltransferase, SNAT, melatonin, abiotic stress, cotton

## Abstract

Serotonin N-acetyltransferase (SNAT) is a key enzyme in the biosynthesis of melatonin, and plays an important role in the regulation of melatonin synthesis. The study of SNAT is of great significance to understand the function of melatonin. In this study, we analyzed the structural characteristics, phylogenetic relationship, gene structure, expression pattern, evolutionary relationship and stress response of the members of the SNAT gene family in upland cotton through bioinformatics. A putative Serotonin n-acetyltransferase gene *GhSNAT3D* was identified, and preliminarily function of *GhSNAT3D* was verified by virus-induced gene silencing. We identified a total of 52 *SNAT* genes in the whole genome of *G. hirsutum*, and part of the *GhSNAT*s were regulated by exogenous melatonin. The content of melatonin, antioxidant enzyme activity and Ca^2+^ content of *GhSNAT3D* gene silenced plants decreased, and the salt tolerance of *GhSNAT3D* gene silenced plants was reduced. Exogenous melatonin supplementation restored the salt tolerance of *GhSNAT3D* gene silenced plants. *GhSNAT3D* may interact with *GhSNAT25D* and *ASMT* to regulate melatonin synthesis. This study provided an important basis for further study on the regulation of melatonin in cotton against abiotic stress.

## Introduction

A hormone from the pineal gland of cattle in 1958 that caused frog skin to fade was named melatonin (N-acetyl-5-methoxy-tryptophane) (Lerner et al., 1958). Melatonin is a tryptophan - derived metabolite widely found in plants and animals (Kanwar et al., 2018). Melatonin as an antioxidant has been reported to control ROS and lipid peroxidation in animal tissues (Reiter et al., 2014). In 1995, two groups of scientists simultaneously discovered the presence of melatonin in vascular plants, opening the door to the study of plant melatonin (Dubbels et al., 1995; Hattori et al., 1995), subsequent studies have shown that melatonin is widely present in plants (Blask et al., 2004). Melatonin is involved in a variety of physiological functions in plants, including seed germination, growth, rooting, photosynthesis and stress resistance. It is considered to be a multi-regulatory molecule and may play a major role in plant regulation (Arnao and Hernandez-Ruiz, 2019).

Cotton, as a kind of cash crop, is often affected by some external environment in the process of growth and development, such as drought, salinity, high temperature, low temperature and so on. The harsh external environment affects the growth of cotton, reduces the yield and fiber quality of cotton. It is of great significance to improve the research of cotton resilience. As a signal molecule, melatonin promotes Ca^2+^ release by regulating phosphatidylinositol signal system and improves salt tolerance of cotton (Zhang et al., 2021a). Melatonin is involved in regulating plant responses to various biological and abiotic stresses, such as enhancing plant resistance to drought, cold, heat, osmotic stress, herbicide, ultraviolet radiation and oxidative stress (Zhang et al., 2015). Since melatonin is involved in the developmental process and stress response of many plants, the exploration of its function in plants has become a rapidly developing field. The data confirms that melatonin is a universal and common antioxidant compound that can interact with a variety of ROS and RNS and is a key factor in the stability of biofilms, especially mitochondrial membranes (Galano et al., 2011). The reported functions of melatonin in plants include regulating growth and development (Lv et al., 2021), regulating circadian rhythm (Chang et al., 2021), scavenging of reactive oxygen species and improving plant resistance to stress. Melatonin is used in plants as a protective agent against a variety of stress situations (biological and abiotic) (Arnao and Hernández-Ruiz, 2014). As a kind of stress hormone, melatonin can improve the stress resistance ability of plants through its own synthesis or exogenous application.

In plants, tryptophan generated melatonin *via* four sequential enzymatic reactions (Park et al., 2012; Tan and Reiter, 2020). The four enzymes were TDC (tryptophan decarboxylase) (Luca and Brisson, 1989), T5H (tryptamine 5-hydroxylase) (Fujiwara et al., 2010), SNAT (serotonin *n*acetyltransferase) (Lee et al., 2014), ASMT (*N-*acetylserotonin methyltransferase) (Kang et al., 2011). In addition, COMT (Caffeic acid *o*-methyltransferase) also has ASMT activity reported in *Arabidopsis thaliana* (Byeon et al., 2014). SNAT is the third key enzyme in the melatonin synthesis pathway, responsible for the production of N-acetylserotonin (Kang et al., 2010), and plays an indispensable role in the study of melatonin function. Transgenic rice expressing human 5-hydroxytryptamine N-acetyltransferase gene (*SNA*/*AANAT*) showed high levels of melatonin and chlorophyll under low temperature stress, suggesting that melatonin plays a role in cold stress tolerance (Kang et al., 2010). Compared with wild type, over expression of *VvSNAT2* in transgenic *Arabidopsis thaliana* increased melatonin and chlorophyll accumulation and enhanced powdery mildew resistance (Yu et al., 2019). Serotonin N-acetyltransferase1 (*OsSNAT1*) is overexpressed in transgenic rice and can resist cadmium, aging and increase production (Lee and Back, 2017). Overexpression of *VvSNAT1* in Arabidopsis enhances salt tolerance by reducing accumulation of malondialdehyde (MDA) and hydrogen peroxide (HO) (Wu et al., 2021). Compared with wild type rice and single control rice, RNA interference inhibited seedling growth retarded with two SNAT genes and significantly decreased melatonin and seed life span (Hwang and Back, 2020). Therefore, studying the function of *SNAT* in cotton is of great significance to explore cotton melatonin and improve cotton stress resistance.

In this study, we identified members of the SNAT family in four cotton species, analyzed the evolutionary relationship, gene structure and expression pattern of the members of the SNAT family in cotton using bioinformatics methods. And we cloned a putative *SNAT* in upland cotton, and preliminarily explored its function by virus-induced gene silencing. *GhSNAT3D*-silenced plants reduced their melatonin level, antioxidant enzyme activity and salt tolerance, while exogenous melatonin supplementation alleviated the negative effects of reduced melatonin level.

## Materials and Methods

### Identification of SNAT Genes

In order to identify members of the SNAT gene family, protein sequences and genomes of upland cotton were downloaded from the cotton database Gossypium Resource And Network database (http://grand.cricaas.com.cn) and CottonFGD(https://cottonfgd.org) (Zhu et al., 2017). The conserved domain of rice SNAT(AK059369) protein sequence was found to be Acetyltransf_1 by Pfam database (https://pfam.xfam.org/) analysis (Kang et al., 2013). Using the hidden Markov model of Acetyltransf_1 as a query file, genes with the conserved domain of Acetyltransf_1 in upland cotton genome were screened by the local software HMMER as candidate genes of SNAT gene family. Based on the conserved domain of SNAT protein, Acetyltransf_1(PF00583) was further analyzed by using the Pfam database, and genes with incomplete domain were manually deleted. Based on the location of the gene on the chromosome, we renamed the gene as *GhSNAT1A*-*GhSNAT24A*, *GhSNAT1D*-*GhSNAT28D*. The physicochemical properties of *GhSNAT* genes were analyzed using the online tool Expasy-Protparam (https://web.expasy.org/protparam/) (Gasteiger et al., 2005). In order to understand the subcellular localization of SNAT protein, we used online websites WOLF-PSORT (https://wolfpsort.hgc.jp/) for prediction.

### Phylogenetic Analysis

In order to study the evolutionary relationship between SNAT genes, the protein sequence of upland cotton SNATs was input into software MEGA7, and ClustalW was used for multiple sequence alignment. The intraspecific evolutionary tree was constructed by neighborhood method, and the specific parameters were as follows: Bootstrap replication: 1,000, model/method: P-disrance, and all/Missing Data Treatment: Partial deletion.

In order to explore the evolutionary relationship of SNAT among different species, the online Phytozome V12.1 database was used to download the protein sequences of *Theobroma cacao*, *Arabidopsis thaliana*, *Oryza sativa*, *Populus trichocarpa*, *Vitis vinifera*, *Glycine max*, *Zea mays*. The homologous sequences of SNAT in these species were obtained by using the above methods. Protein sequences from four cotton species (*Gossypium arboretum*, *Gossypium raimondii*, *Gossypium hirsutum*, *Gossypium barbadense*) and members of the SNAT family of *Theobroma cacao*, *Oryza sativa*, *Arabidopsis thaliana* were input into MEGA7. ClustalW was used for multiple sequence alignment and Maximum Likelihood method was used to construct interspecific evolutionary tree. Parameter Settings were as follows: bootstrap replication 500, model/method: JTT + G, and all/Missing Data Treatment: Partial deletion (Kumar et al., 2016).

### Analysis of Gene Structure and Motif Composition

The gene structure of GhSNAT members was obtained by using the annotation file of whole genome of upland cotton downloaded from online cotton database CottonFGD. Online software MEME was predicted the motif of genes (http://meme-suite.org/tools/meme), and the parameters were as follows: the maximum number of motif of each gene is 20, and other parameters are set by default. The evolutionary relationship, gene structure and motif composition of SNAT were analyzed using TBtools (Chen et al., 2020).

### Chromosome Localization and Gene Replication

The whole genome annotation files of four cotton species were downloaded from cotton database CottonFGD (https://cottonfgd.org/about/download/annotation), and gene replication events occurred in SNATs were analyzed by MCScanX software. The chromosome positions and gene replication of SNATs members of four cotton species were visualized by TBtools software (Chen et al., 2020).

### Expression Pattern and Promoter Analysis

The 2000 bp DNA sequence of the upstream region of GhSNAT was obtained from CottonFGD database (https://cottonfgd.org) as the promoter (Martinez and Chrispeels, 2003). We used PlantCARE (http://:/bioinformatics.psb.ugent.be/webtools/PlantCARE/html/) to predict *cis*-regulatory elements in the GhSNATs gene promoter region. *Cis*-acting elements related to plant hormones and abiotic stress were selected for further analysis and plotted by software TBtools (Chen et al., 2020).

In order to study the expression patterns of GhSNAT gene family, we obtained the expression levels of these genes under cold, heat, salt, PEG stress and the expression levels of these genes in roots, stems and leaves by Gossypium Resource And Network database online (http://grand.cricaas.com.cn/page/tools/expressionVisualization). Heat maps were generated using TBtool software using fragment number per kilobile exon (FPKM) (Chen et al., 2020).

### Collinearity Analysis of SNATs

In order to study the evolutionary relationship of SNATs in four cotton species, MCScanX software was used to analyze the collinearity of repeated gene pairs in *Gossypium arboretum*, *Gossypium raimondii*, *Gossypium hirsutum*, *Gossypium barbadense* (Wang et al., 2012), the software TBtools was used to visualize the results (Chen et al., 2020).

### Selective Pressure Calculation

To determine selection pressure, Ka (non-synonymous substitution) and Ks(synonymous substitution) rates of duplicate genes were calculated using TBtools software (Chen et al., 2020).

### Plant Growth and Treatment

Using upland cotton cultivar Zhong 9807 as experimental material, the seeds were sown on the medium of sand and vermiculite 1:1.5, and grew in an indoor incubator at 25°C for 16 h in the day and 8 h at night. In order to explore the effect of increasing melatonin level on *GhSNAT*s expression, cotton seedlings at three-leaf stage were treated with 20 µM melatonin and sprayed on leaves once a day for three consecutive days. The seedlings were grown in an indoor incubator at 25°C for 16 h during the day and 8 h at night (Zhang et al., 2021a). Melatonin treatment with 0 µM concentration was used as control. The cotton seedlings treated with melatonin and the control group were treated with NaCl solution at 100 mM concentration for 12 h and sampled respectively. The three biological replicates were performed.

### GhSNATs Expression Was Detected by qRT-PCR

Total RNA was extracted by EASYspin Plus Plant RNA rapid isolation kit (Aidlab Co., LTD, Beijing, China). The pure RNA was reverse-transcribed using HiScript Ill RT SuperMix for qPCR (+gDNA wiper) (Vazyme Biotech Co., LTD, Nanjing, China) according to the manufacturer’s instructions. Gene-specific primers were designed using Primer Premier 5 software (Supplementary Table S1). qRT-PCR assays were performed on the Bio-Rad 7,500 fast fluorescence quantitative PCR platform with TransStart^®^ top green qPCR supermix (TransGene Biotech Co., LTD, Beijing, China) in accordance with the manufacturer’s protocol, three biological replicates, 2^−ΔΔCt^ methods were used to measure relative gene expression levels (Livak and Schmittgen, 2002). The internal control was GhUBQ7 (Tan et al., 2013), which was expressed stably in cotton plants and was not affected by treatment or genotype.

### VIGS Technology Silenced *GhSNAT*

In order to investigate whether the content of endogenous melatonin in cotton affected the salt tolerance of cotton, the SNAT gene was silenced by virus-induced gene silencing. We used the published protein sequence of rice *SNAT*(AK059369) as the query sequence (Kang et al., 2013), the putative *GhSNAT*(GH_D02G1113.1) in cotton was obtained by comparing the cotton genome with local software BLAST, which was named *GhSNAT3D* in this study. VIGS vector was stored in our laboratory as pYL156 vector, BamHI and SacI restriction endonuclease were selected for double digestion, and online tool SGN-VIGS (https://vigs.solgenomics.net/) was used to design silent fragments with a length of about 300bp. The Infusion primer for *GhSNAT* was manually designed, and the primer for *GhSNAT* silent fragment was as follows: forward primer, 5′-TAG​AAG​GCC​TCC​ATG​GGG​ATC​CGA​ATT​TGT​GCT​TGT​TGA​AAA​GTC​TCA-3′ reverse primer, 5′-TGC​CCG​GGC​CTC​GAG​ACG​CGT​GAG​CTC​AGG​ATC​AAC​AAG​AAC​ATC​CCA​GA-3′. Using cDNA from cotton leaf tissue as template, the silent fragment was amplified and the VIGS expression vector pYL156:GhSNAT was constructed by in-fusion technique. The constructed expression vector was transformed into *EScherichia coli*. After correct sequencing, the expression vector was transformed into Agrobacterium tumefaciens by freeze-thaw method. The virus-mediated gene silencing (VIGS) system consisted of recombinant vector, negative control pYL156, positive control pYL156:PDS and helper vector pYL192. Therefore, the correctness of the whole system was judged by observing whether the true leaves of pYL156:PDS VIGS turned white. Prepare cotton seedlings for infection after cotyledons flatten, and water them thoroughly the night before infection. Make an incision in the lower *epidermis* of the cotyledon with a needle, and inject the bacteria into the lower *epidermis* of the cotyledon with a syringe until the bacteria fill the whole cotyledon. After the injection, the cotton seedlings were sheltered from light for 24 h and then cultured normally. Cotton was treated with 100 mmol L^−1^ NaCl when it reached the three-leaf stage.

### Detection of Melatonin Content

We took samples for the determination of endogenous Melatonin content, and measured the endogenous Melatonin content by Plant Melatonin (MT) ELISA Kit (Ziker, ZK-P7490, Shenzhen, China). This kit is intended for use only with a one-step sandwich ELISA kit. The 0.1 g sample was mashed with proper amount of normal saline, centrifuged at 3,000 rpm for 10 min, and the supernatant was taken. Melatonin was detected according to the Plant MT ELISA Kit, and each sample had three biological replicates.

### Detection of Antioxidant Enzyme Activity

Peroxidase (POD) activity and superoxide dismutase (SOD) activity were determined by using POD activity detection kit (Sinobestbio, YX-W-A502, Shanghai, China) and superoxide dismutase (SOD) activity assay kit (Sinobestbio, YX-W-A500-WST-8, Shanghai, China). About 0.1 g tissue was weighed and 1 ml extract was added for ice bath homogenization. Centrifuged 8,000 *g* at 4°C for 10min, supernatant was taken and placed on ice for determination according to the instructions, with three biological replicates for each sample.

### Detection of Ca^2+^ Content Detection

The determination of Ca^2+^ content refers to EDTA titration method in GB 5009.922016 “Determination of Calcium in Food of National Standard for Food Safety”. Samples that needed to be assayed for Ca^2+^ content were cleaned by ddH_2_O, placed in an oven, oven dried at 110°C for 10 min, 80°C until constant weight, accurately weigh 0.2 g of sample into a graduated digestion tube, add 10 ml 10% nitric acid, and digest in an adjustable electric furnace (reference conditions: 120°C/0.5 h to 120°C/1 h, increase to 180°C/2 h to 180°C/4 h, and increase to 200–220°C). The digestion solution appeared colorless and transparent or slightly yellow. Constant volume to 25 ml with water after cooling, then dilute as needed for the actual assay, and add a volume of lanthanum solution (20 µg/L) to the dilution to a final concentration of 1 μg/L and mix for further use, this being the sample to be tested. Pipette 1 ml of the sample to be tested and a blank into a test tube and add 1 drop of sodium sulfide solution (10 g/L), 0.1 ml of sodium citrate solution (0.05 mol/L), 1.5 ml of potassium hydroxide solution (1.25 mol/L), and 3 drops of calcium red indicator. Titrate immediately in a 10 fold dilution of EDTA solution until the indicator changes blue from purple red and record the volume of EDTA solution consumed with a 10 fold dilution.

### GhSNAT Protein Interaction Network Prediction

In order to explore the interaction network of GhSNAT protein, protein sequences of *GhSNAT3D* were compared to *Arabidopsis thaliana* to obtain the homologous genes of *GhSNAT3D* in Arabidopsis. STRING database (https://string-db.org/) was used to construct the interaction network and analyze the functions of GhSNAT protein.

### Statistical Analysis

The GraphPad Prism 8.0 software was employed to analysis (ANOVA) the results. Duncan’s Multiple Range Test was used to compare the least signifcant difference of means (*p* < 0.05).

## Results

### Identification of *GhSNAT* Family Members

The latent Markov model of Acetylation transf_1 was used as a query file, and genes with conserved domain of Acetylation transf_1 were selected as candidate genes of SNAT gene family in upland cotton by HMMER software. Pfam database was used for further analysis, and genes with incomplete domain were removed manually. We renamed the genes *GhSNAT1A*-*GhSNAT24A*, *GhSNAT1D*-*GhSNAT28D* based on their location on the chromosome. We then analyzed and predicted the physical properties of these genes, including ID, isoelectric point, protein molecular weight, protein length and subcellular localization (Supplementary Table S2). A total of 52 SNAT genes were identified in the whole upland cotton genome, which encoded protein sequences ranging from 157 (*GhSNAT1A*, *GhSNAT1D*) to 610 (*GhSNAT4A*) amino acids. The isoelectric point ranged from 5.083 (*GhSNAT9A*, *GhSNAT10D*) to 10.08 (*GhSNAT25D*), and the molecular weight ranged from 17.718 (*GhSNAT1D*) kDa to 67.138 (*GhSNAT16A*) kDa. Subcellular localization predicted 21 genes in cytoplasm, 3 genes in cytoskeleton, 2 genes in mitochondria, 20 genes in chloroplast, 6 genes in nucleus.

### Phylogenetic Analysis of *SNAT*s

**FIGURE 1:**
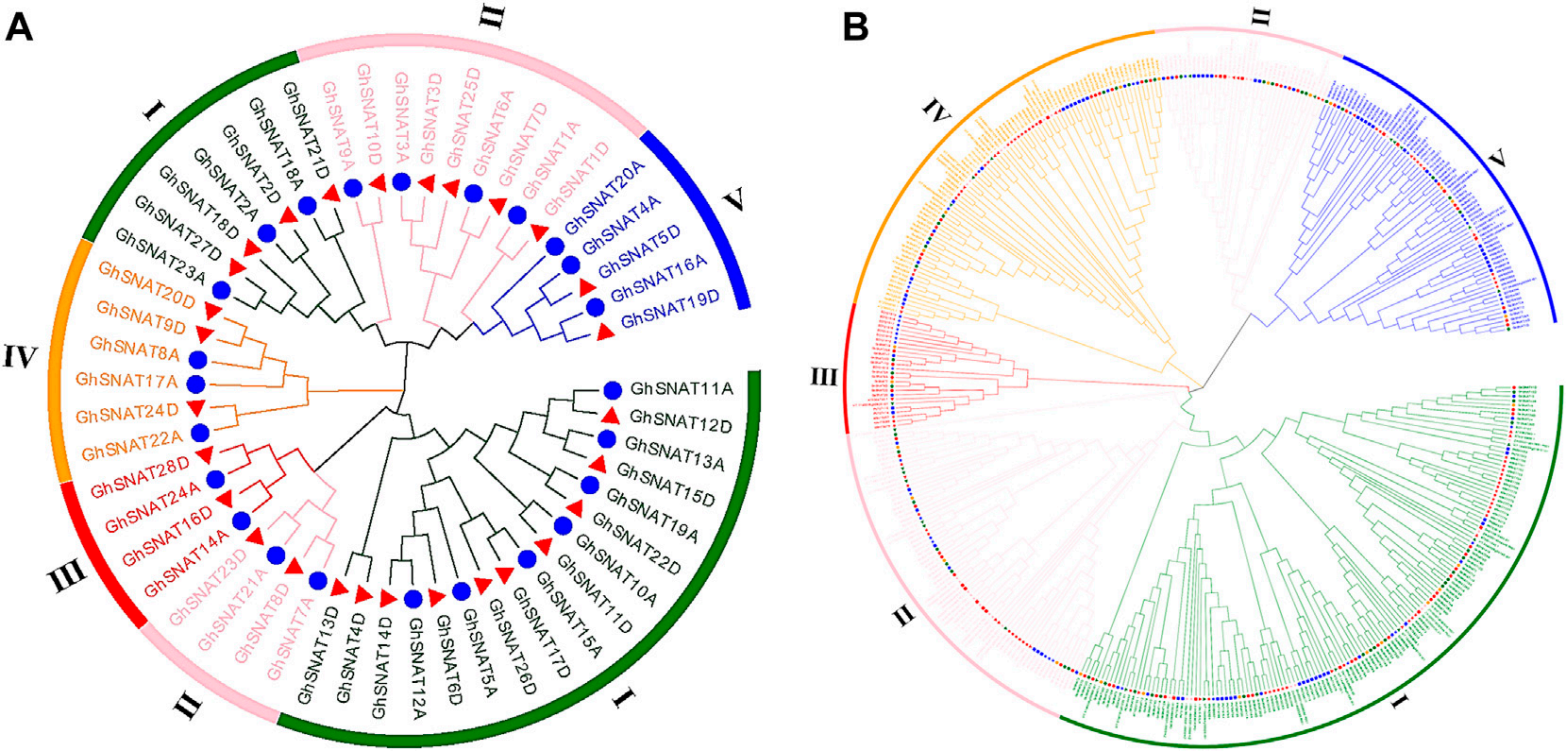
Two unrooted phylogenetic trees were constructed by the MEGA7, Neighborhood method was used to draw the evolutionary tree of GhSNATs family, and Maximum Likelihood method was used to draw the interspecific evolutionary tree of SNATs. **(A)**: Evolutionary analysis of members of the SNAT family *Gossypium hirsutum*; **(B)**: Phylogenetic relationships of 468 SNAT proteins from cotton, *Theobroma cacao*, *Arabidopsis thaliana*, *Oryza sativa*, *Populus trichocarpa*, *Vitis vinifera*, *Glycine max*, *Zea mays*.

In order to further study the evolutionary relationship of SNAT in upland cotton, we constructed an evolutionary tree of intraspects-specific *SNAT* genes in upland cotton (Figure 1A). According to the evolutionary relationship of genes and protein structure characteristics, the *SNAT* genes in upland cotton can be divided into five branches. Branch Ⅰ contains 24 genes and branch Ⅱ contains 13 *GhSNAT*s, branch Ⅲ contains 4 *GhSNAT*s, branch Ⅳ has 6 *GhSNAT*s, and branch Ⅴ has 5 *GhSNAT*s. In the phylogenetic tree, two genes gather together to form gene pairs, which form 22 gene pairs in total. In each pair, one gene comes from the A subgenome and one from the D subgenome.

To study the evolutionary relationships of SNAT in plants, 468 protein sequences from upland cotton, island cotton, Asiatic cotton, Raymond’s cotton, cocoa, Arabidopsis, rice, soybean, grape, poplar and maize (27 from *Gossypium arboretum*, 59 from *Gossypium barbadense*, 52 from *Gossypium hirsutum*, 26 *Gossypium raimondii*, 33 from *Arabidopsis thaliana*, 31 from *Oryza sativa*, 48 from *Theobroma cacao*, 49 from *Zea mays*, 102 from *Populus trichocarpa*, 114 from *Glycine* max and 36 from Vitis vinifera) were constructed to study the relationship between SNAT genes (Figure 1B). The SNAT proteins of these species were distributed in almost every clade. The phylogenetic tree was divided into five branches randomly distributed. Among these clades, clade Ⅲ had the least number of members (26), clade Ⅰ had the most members (141), clade Ⅱ, Ⅳ and Ⅴ contained 111, 85 and 70 genes, respectively. It should be noted that in Arabidopsis, rice and cacao, SNAT proteins of four cotton species all have corresponding homologous genes in clade Ⅰ - Ⅴ, indicating that the SNAT proteins of these plants are closely related in evolution. In phylogenetic trees, we found that the *GhSNAT* gene pairs and *GbSNAT* gene pairs were always clustered together, which could serve as evidence of gene duplication. At the same time, SNAT proteins of tetraploid cotton (*Gossypium hirsutum*, *Gossypium barbadense*) and diploid cotton (*Gossypium arboretum*, *Gossypium raimondii*) were clustered together, confirming that upland cotton and island cotton were the result of hybridization between *Gossypium arboretum*, and *Gossypium raimondii*.

### Correlation Analysis of *GhSNAT* Gene Structure and Motif Composition

**FIGURE 2:**
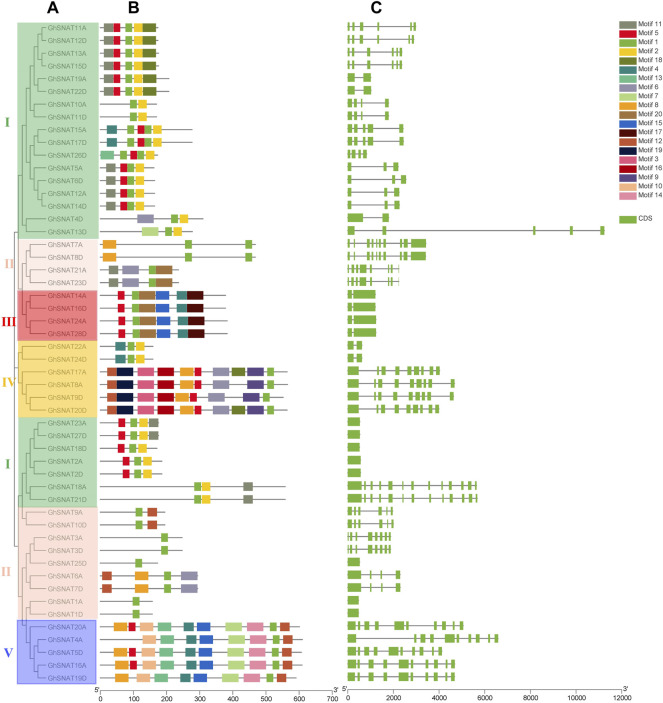
The evolutionary relationship of *GhSNATs* in *Gossypium hirsutum*, the association analysis of motif composition and gene structure. **(A)**: The phylogenetic tree of *GhSNAT*s; **(B)**: The motif composition of *GhSNAT*s; **(C)**: The genetic structure of *GhSNAT*s.

To further understand the possible structural evolution of *GhSNAT*s, we constructed a phylogenetic tree, gene structure, and motif association analysis of *GhSNAT* members (Figure 2). We constructed a phylogenetic tree using the protein sequences of *GhSNAT* members, obtained the gene structure of *GhSNAT* members from the whole genome annotation file of upland cotton, submitted the protein sequences of *GhSNAT* members to the online tool MEME for conservative motif prediction, and used TBtools for association analysis.

From gene structure and phylogenetic tree results, we can see that similar genes clustered together in the same set of phylogenetic trees. The number of exons in each gene ranged from 1 to 13. In most cases, two genes in the gene pairs had similar exon-intron structure and length, but some gene pairs were different in structure and length, such as *GhSNAT4D*/*GhSNAT13D* and *GhSNAT4A*/*GhSNAT5D*. There were 8 genes with only one exon and 2 genes with 13 exons. Different clades have different numbers of exons, but most *GhSNAT* members of the same clades share the same exon-intron structure. We found that *GhSNAT* gene structure is strongly related to phylogeny on an evolutionary basis. The motifs of each protein vary from 1 to 10. In addition, two genes in most gene pairs have the same motif composition, meaning that they are functionally similar at the protein level. And all *GhSNAT* proteins contained Motif1, a conserved domain shared by the *GhSNAT* family. Clade I mainly consists of motifs 1 and 2, and most members in clade I also contained motif5 and 11. Most of the *GhSNAT* motif compositions in clade Ⅱ were irregular, but all have motif1. The motif composition of clade III was more conserved and consists of motif5, 1, 15, 20, 4, 17. Clade Ⅳ mainly consists of motif12, 19, 3, 16, 8, 5, 6, 9, and 1, but *GhSNAT22A* and *GhSNAT24D* consist of motif4, 1, and 2; most members of clade V consist of motif10, 13, 4, 15, 7, 14, 1, 16. Interestingly, the *GhSNAT13D*/*GhSNAT4D* genes were not only different in gene structure, but also in motif composition, one of which was composed of Motif6, 1, 2, and one of which was composed of motif7, 1, 2. We speculated that it may have been mutated during evolution leading to changes in gene structure and function.

### Chromosome Location and Gene Replication of *SNAT*s

**FIGURE 3:**
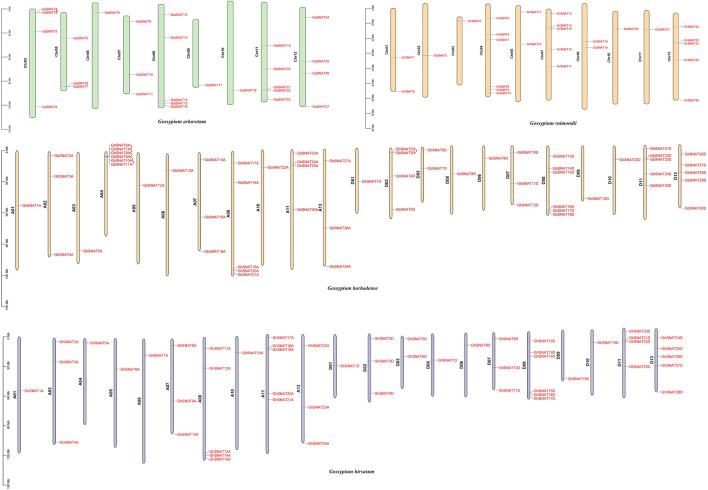
Chromosome localization and gene replication of *SNAT*s. *SNAT*s were distributed in *Gossypium arboretum*, *Gossypium Raimondii*, *Gossypium hirsutum*, and *Gossypium barbadense*, and tandem repeats occurred in the gene pairs connected by wires during evolution.

In order to study the chromosome distribution and gene replication of members of the SNAT gene family, we mapped the physical locations of these genes on cotton chromosomes (Figure 3). 164 *SNAT*s were located on specific chromosomes of four cotton species. In *Gossypium hirsutum*, 52 genes were mapped onto 21 chromosomes, with uneven distribution. The number of *SNAT* genes on each chromosome was between 1-5. There were 24 genes in A subgenome and 28 genes in D subgenome. Chromosomes A03, A09, A12, D04 and D12 did not have SNAT. The number of genes on chromosomes A01/D01, A02/D02, A05/D05, A06/D06, A07/D07, A08/D08, A10/D10 was consistent, while the number of genes on other A/D chromosomes was different, which might be caused by the duplication or loss of *SNAT* members in the process of evolution. Interestingly, *GhSNAT*s did not undergo tandem repetition during evolution, and fragment replication was the main mode of gene amplification.

In *Gossypium barbadense*, 59 genes were distributed on 22 chromosomes, with 1-6 SNATs on each chromosome, 29 *SNAT*s on the A subgenome and 30 *SNAT*s on the D subgenome. There were 6 *SNAT*s on chromosomes A04 and D08, but no *SNAT*s on chromosomes A09, A12, D04 and D12, suggesting that gene loss may be one of the reasons for species evolution. Although the number of SNAT in A/D subgenome was similar, the distribution of these genes in chromosomes was not corresponding. The number of genes on chromosome A01/D01, A05/D05, A06/D06, A07/D07, A10/D10 was the same, while the number of genes on other A/D chromosomes was different. In *Gossypium barbadense*, there were five tandem replication on chromosome A04 and one tandem replication on chromosome D02. Tandem replication was also one of the driving forces for the amplification of *GbSNAT*s in evolution.

In Gossypium arboretum, 27 *GaSNAT*s were unevenly distributed on 9 chromosomes, wherein chromosome Chr06, Chr09 and Chr10 have 1 *GaSNAT* respectively, and chromosome Chr08 and Chr11 have at most 5 *GaSNAT*s. No genes were distributed on chromosome Chr01, Chr02, Chr04, and Chr12, and a tandem replication occurred on chromosome Chr03. In *Gossypium raimondii*, 26 *GrSNAT*s were distributed unevenly on 10 chromosomes, and no *GrSNAT*s were distributed on chromosomes Chr06, Chr08, and Chr12. In *Gossypium raimondii*, *GrSNAT*s did not undergo tandem replication during evolution.

### *GhSNAT* Expression Pattern and Promoter Analysis

**FIGURE 4:**
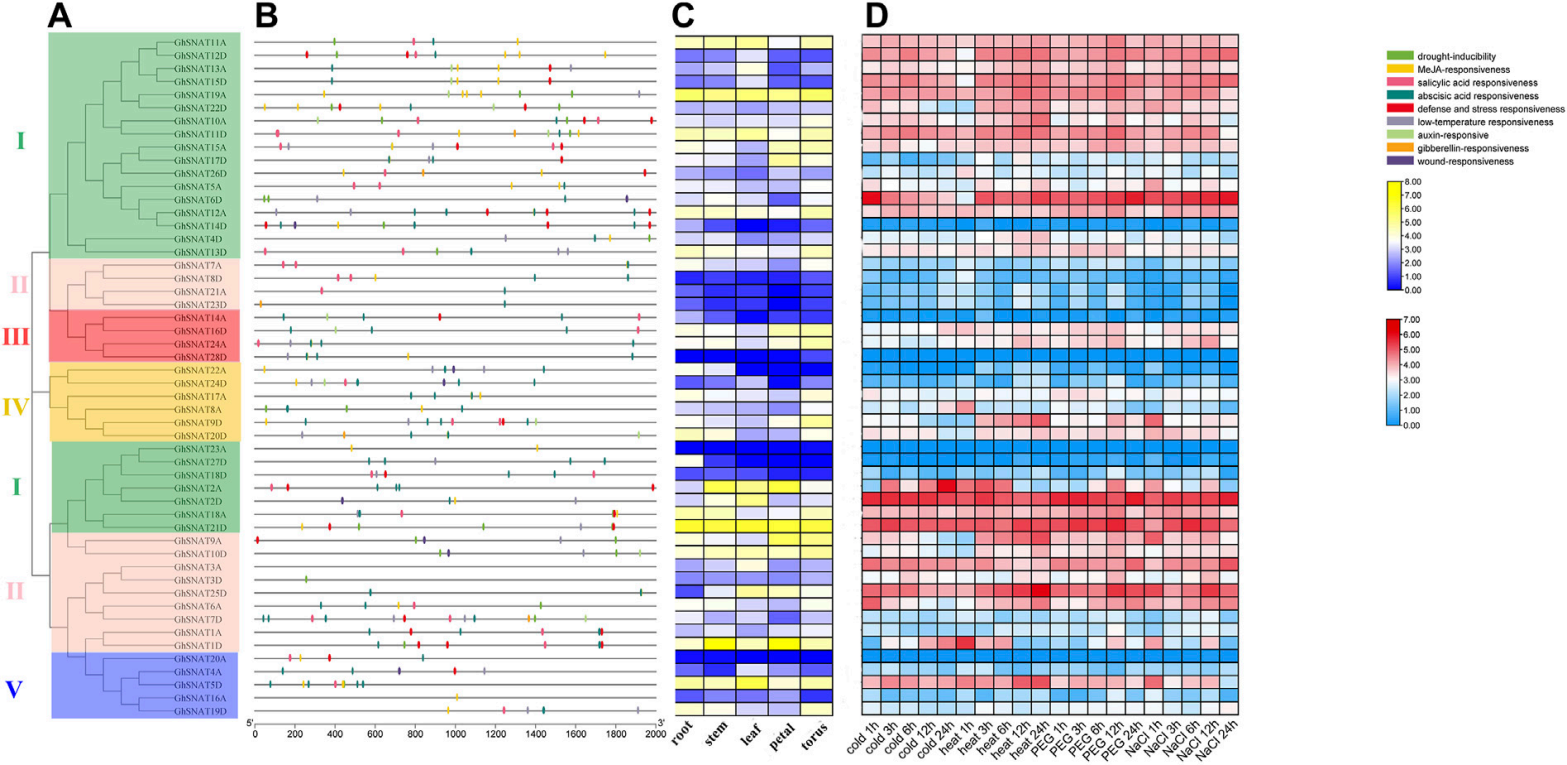
Expression pattern and promoter analysis of the *SNAT* gene family in *Gossypium hirsutum*. **(A)**: The phylogenetic tree of *GhSNAT*s; **(B)**: The promoter analysis of *GhSNAT*s; **(C)**: The organizational expression of *GhSNAT*s; **(D)**: The expression patterns of *GhSNAT*s under abiotic stress.

Promoters can interact with transcription factors to control the onset and degree of gene expression. *Cis*-acting elements are located in the promoter region of genes and can be used as a reference for stress response and tissue specificity in different environments. To understand the response mechanism of *GhSNAT* to abiotic stresses, we obtained FPKM of *GhSNAT*s from Gossypium Resource And Network database online to analyze the expression patterns of these genes under various stresses (cold, heat, salt, and PEG). Phylogenetic tree analysis, promoter analysis and expression heat map association analysis were performed (Figure 4). Therefore, the analysis of *GhSNAT* promoter region was helpful to explore the potential function of genes. *Cis*-acting elements played an important role in stress response. We used 2000 bp DNA sequence of the upstream region of *GhSNAT* as the promoter, and predicted *cis*-acting elements of the promoter region of *GhSNAT* gene by using the online tool PlantCARE. A large number of *cis*-acting elements were detected in the promoter region, and *cis*-acting elements related to plant hormones and abiotic stresses were selected for further analysis.

In the plant hormone response category, the selected elements are abscisic acid response element, salicylic acid response element, gibberellin response element, MeJA response element and auxin response element; the elements responding to abiotic stress include defense and stress response element, wound response element, drought-inducibility element and low-temperature response element. More than half of *GhSNAT* members had MeJA-responsive, abscisic acid responsiveness, salicylic acid responsiveness, 41 genes contained abscisic acid responsiveness elements, 8 genes contained wound responsiveness, 5 genes contained gibberellin-responsive, and 13 genes contained auxin-responsive. There were 20 *GhSNAT*s with defense and stress responsiveness elements, and there were 19 *GhSNAT*s with drought-inducibility elements. 23 *GhSNAT*s contained low-temperature responsive elements. We speculated that these genes are involved in abiotic stress responses along with hormo responses.

A total of 52 FPKM of *GhSNAT*s were obtained from Gossypium Resource And Network database, and heat maps were made based on the expression levels of these genes under cold, heat, salt and PEG stresses. The results showed that only a few *GhSNAT*s, such as *GhSNAT14D*, *GhSNAT28D*, *GhSNAT23A*, *GhSNAT27D*, *GhSNAT20A* were not differently expressed under various abiotic stresses. Most of the genes were strongly induced and differentially expressed under various stresses. We found that most of the genes from the same clade had the same expression pattern. Interestingly, some genes were induced by specific stresses, such as *GhSNAT1D*, which was strongly induced under cold stress but not under other stresses; *GhSNAT9D* was only induced by heat stress. We counted the number of differentially expressed *GhSNAT* genes under different stresses, including 29 differentially expressed *GhSNAT*s under cold treatment, 35 differentially expressed *GhSNAT* genes under high temperature treatment, 40 differentially expressed *GhSNAT* genes under PEG treatment and 37 differentially expressed *GhSNAT* genes under salt stress. The expression levels of *GhSNAT* genes changed under different stress conditions, suggesting that *GhSNAT* members were involved in the regulation of abiotic stress.

Meanwhile, to explore the tissue specificity of *GhSNAT*s, we obtained FPKM of *GhSNAT*s expression in different tissues (root, stem, leaf, petal, torus) from Gossypium Resource And Network database And visualized the data by using TBtools. In the GhSNAT family, 7 genes (*GhSNAT8D*, *GhSNAT21A*, *GhSNAT23D*, *GhSNAT28D*, *GhSNAT23A*, *GhSNAT18D*, *GhSNAT20A*) showed low expression in different tissues. *GhSNAT19A* and *GhSNAT21D* were highly expressed in each tissue, and most of the genes were tissue specific, such as *GhSNAT5D*, *GhSNAT11D*, *GhSNAT13A* and *GhSNAT2D* were highly expressed in leaf. *GhSNAT1D* and *GhSNAT2A* were highly expressed in stem and petal. *GhSNAT9D*, *GhSNAT13D* and *GhSNAT12A* were highly expressed in torus.

### Collinearity Analysis

**FIGURE 5:**
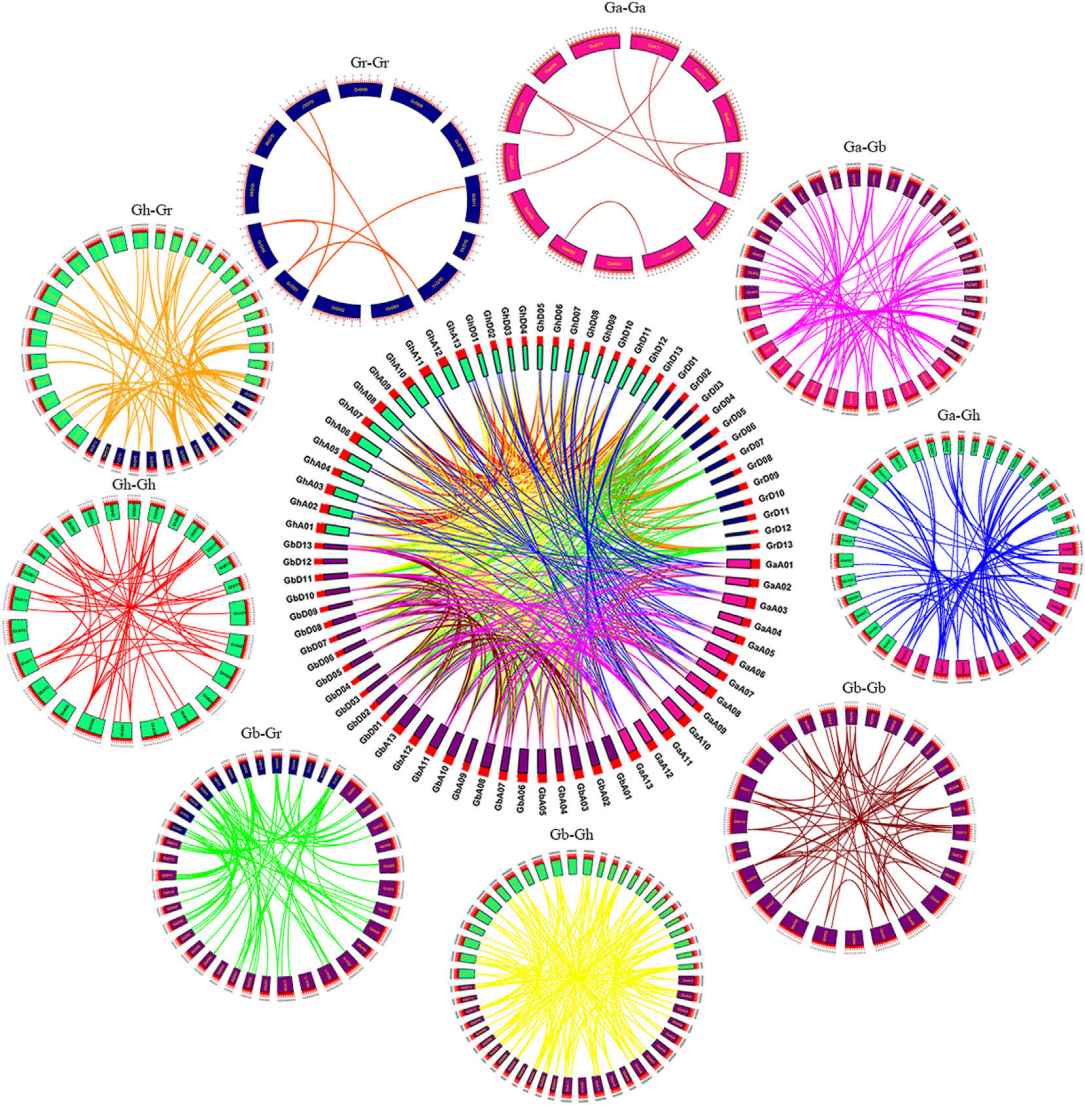
Syntenic relationship of duplicated genes pairs from four cotton species (*G. hirsutum*, *G. barbadense*, *G. arboreum and G. raimondii*). The chromosomal lines in different colors indicate the collinearity area around the *SNAT* genes.

Replication events, including whole genome replication, fragment replication and tandem replication, play an important role in gene amplification. Most plants have experienced ancient genome-wide replication events or polyploidy, and the duplication regions caused by genome-wide replication are usually the duplication of all genes on a large scale, rather than the duplication of a single gene or multiple genes (Malik et al., 2020). In biological evolution, the genome will undergo structural and quantitative changes during the processes of whole genome replication, chromosome recombination, chromosome inversion and translocation (Dujon et al., 2004; Nakatani et al., 2007). Large-scale genome-wide replication (WGD) and small-scale tandem replication and fragment replication between species can be identified from colinear fragments, which can be used as data for species tree inference. Through homology analysis of SNAT genes of four cotton species (*Gossypium arboretum*, *Gossypium raimondii*, *Gossypium hirsutum*, *Gossypium barbadense*), the loci relationships of SNAT genes of four cotton species were visualized (Figure 5). Genes on the same chromosome and adjacent to each other belong to tandem duplications, and the remaining genes from the same genome belong to segmental duplications (Liu et al., 2010), whereas the remaining genes from different genomes and subgenomes belong to the whole genome duplication. Gene duplication events are one of the major contributors to evolutionary dynamics and they have a major impact on genomic rearrangements and expansions (Chothia et al., 2003). We identified homologous gene pairs in GhA, GhD, GbA, GbD subgenomes of two tetraploid cotton and GaA, GrD genomes of two diploid cotton. Collinearity showed that there were several highly conserved loci in the A and D subgenomes of the two tetraploid cotton species, which was also the result of the cross between two tetraploids (*Gossypium hirsutum*, *Gossypium barbadense*) and two diploids (*Gossypium arboretum*, *Gossypium raimondii*).

Genes connected by lines of the same color represent the same gene. In Figure 5, we can see that the GhA/GhD, GbA/GbD subgenomes and many chromosomes in A and D genomes are connected by lines of the same color, that is, the GhA/GhD and GbA/GbD subgenomes have homologous genes of SNAT in A and D genomes. These results indicated that these genomes/subgenomes are evolutionarily related, and most SNAT genes are preserved in polyploid evolution. By comparing the genomes and subgenomes of Ga-Ga, Ga-Gb, Ga-Gh, Gb-Gb, Gb-Gr, Gb-Gh, Gr-Gh, Gr-Gr and Gh-Gh, a total of 608 lineal/parallel homologous gene pairs were identified, and 7 pairs of duplicated genes showed tandem duplication. There were 123 pairs of duplicated genes with fragment replication and the remaining 478 pairs of duplicated genes with whole genome replication. The number of Lineal/Parallel homologous gene pairs in Gh-Ga, Gh-Gr, Gh-Gb, Gb-Gr, and Gb-Ga replicated in the whole genome of *SNAT* gene was 70, 81, 165, 83, and 79, respectively. The colinear gene pairs of Ga-Ga, Gb-Gb, Gh-Gh and Gr-Gr were 8, 57, 52 and 6, respectively. Therefore, we hypothesized that genome-wide replication events and fragment replication events mainly occurred during the evolution of SNAT gene, leading to gene amplification.

### Analysis of Ka/Ks

**FIGURE 6:**
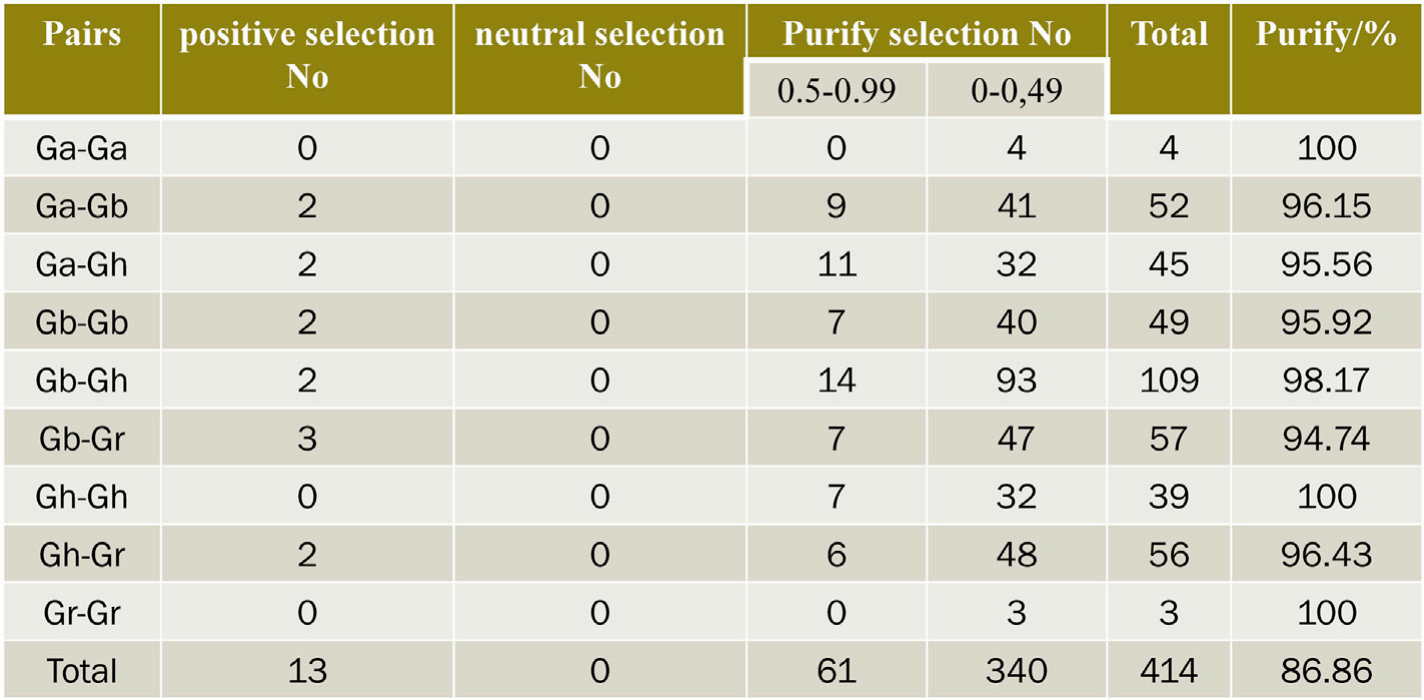
Prediction of no of duplicated gene pairs involved in different combinations from four cotton species.

In order to explore the effect of Darwin’s positive selection on the differentiation of duplicate genes, the present study analyzed the ratio of non-synonymous substitution rate (Ka) to synonymous substitution rate (Ks) in the coding region of all duplicated genes. In genetics, Ka/Ks represents the ratio between the non-synonymous replacement rate (Ka) and the synonymous replacement rate (Ks) of two protein-coding genes. This ratio determines whether there is selective pressure on the protein-coding gene. In general, there are pros and cons (and often cons) of natural selection because non-synonymous substitutions can cause amino acid changes that may alter the conformation and function of proteins and thus cause adaptive changes. TBtools was used to calculate the Ka/Ks ratio of these gene pairs (Figure 6), and the selection pressure of duplicate gene pairs could be inferred from the Ka/Ks ratio. The Ka/Ks values of 414 gene pairs were calculated in four cotton varieties. Generally, Ka/Ks = 1 is considered neutral selection, Ka/Ks < 1 is considered negative selection, and Ka/Ks > 1 is considered positive selection. There were 13 pairs of genes whose Ka/Ks values were all greater than 1, indicating that these genes had undergone positive selection in the evolution process. There were 340 pairs of genes with Ka/Ks values between 0 and 0.5, 61 pairs of genes with Ka/Ks values between 0.5 and 0.99, 96.86% of them with Ka/Ks values less than 1. This suggests that *SNAT*s underwent intense purification and selection during evolution. In Ga-Gb, Gh-Ga, Gb-Gb, Gb-Gh, Gb-Gr and Gh-Gr, the number of gene pairs with Ka/Ks value greater than 1 is 2, 2, 2, 2, 3 and 2, respectively, indicating that these genes have been actively selected in the evolutionary process and have recently undergone rapid evolution. But whether it brings about harmful or beneficial traits remains to be studied.

### Effect of Exogenous Melatonin on *GhSNAT*s Expression

**FIGURE 7:**
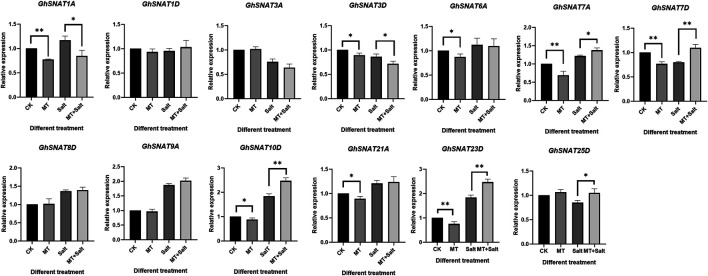
Effect of exogenous melatonin on *GhSNAT*s expression. *p < 0.05 and **p < 0.01.

Exogenous melatonin application has been reported to improve salt tolerance of cotton (Zhang et al., 2021a). In order to explore the effect of exogenous melatonin on *GhSNAT*s expression, we detected the relative expression levels of melatonin on members of cotton *GhSNAT* clade Ⅱ family under salt stress (Figure 7). Under normal growth, some *GhSNAT*s were not affected by exogenous melatonin, such as *GhSNAT1D*, *GhSNAT3A*, *GhSNAT8D*, *GhSNAT9A*, *GhSNAT25D*. The other genes in clade Ⅱ were down-regulated by exogenous melatonin. Under salt stress, *GhSNAT1D*, *GhSNAT3A*, *GhSNAT6A*, *GhSNAT8D*, *GhSNAT9A*, *GhSNAT25D* were not affected by exogenous melatonin. *GhSNAT1A* and *GhSNAT3D* were down-regulated by melatonin. *GhSNAT7A*, *GhSNAT7D*, *GhSNAT10D*, *GhSNAT23D*, and *GhSNAT25D* were up-regulated.

### Preliminary Functional Validation of *GhSNAT*

**FIGURE 8:**
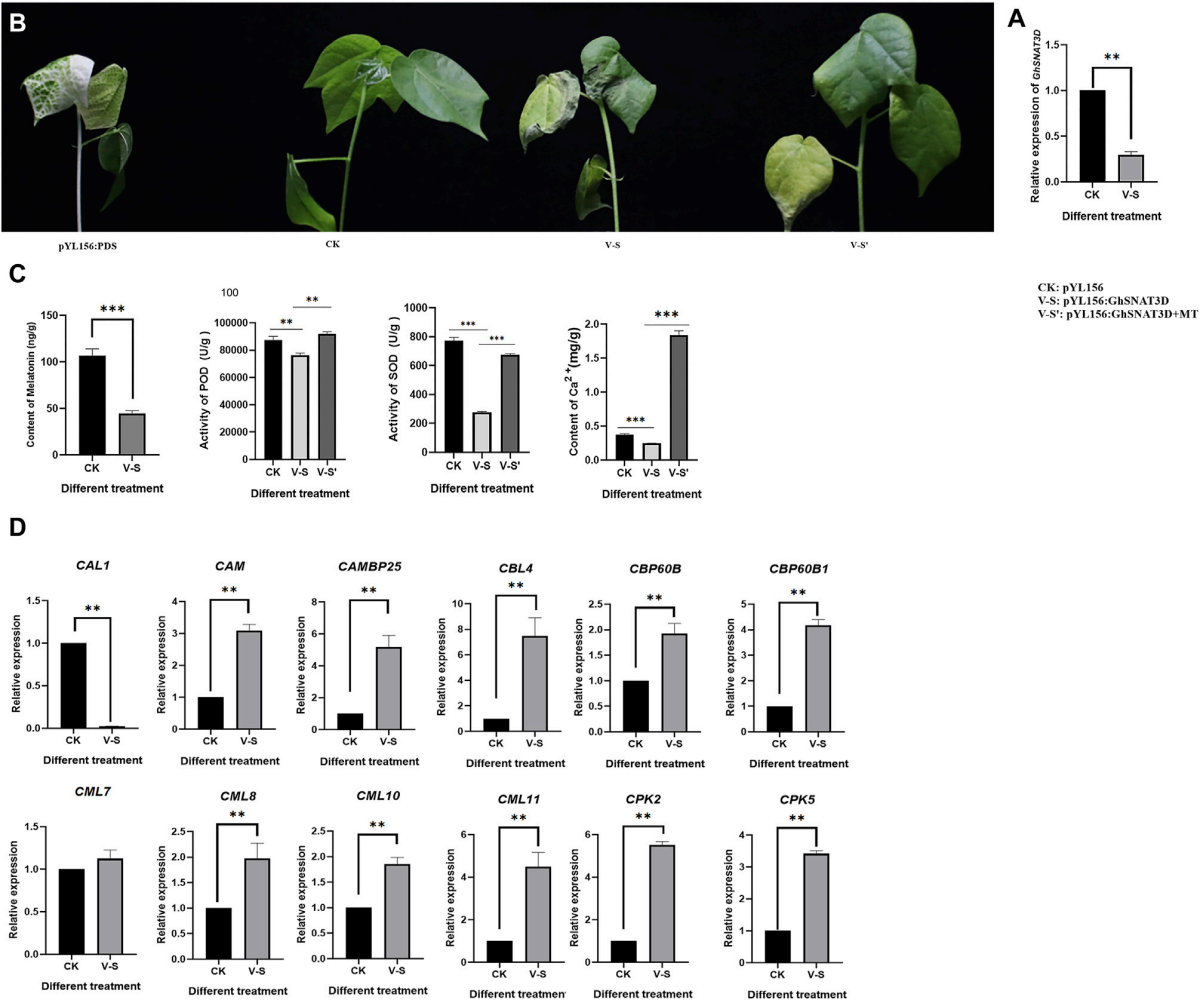
Function verification of *GhSNAT*. **(A)**: Detection of *GhSNAT3D* silencing efficiency. **(B)**: Phenotypic comparison of *GhSNAT3D* silenced plant under salt stress. **(C)**: Detection of melatonin content, POD activity, SOD activity and Ca^2+^ content of *GhSNAT3D* silenced plants. **(D)**: Detection of downstream gene expression of Ca^2+^ signal transduction in *GhSNAT3D* silenced plants. *p < 0.05, **p < 0.01 and ***p < 0.001.

In order to study the function of upland cotton Serotonin N-acetyltransferase (*GhSNAT*), melatonin synthesis gene *GhSNAT3D* was silenced by VIGS technique. PYL156:PDS can change the leaf from green to white. The success of the experiment can be judged by observing whether the leaves of pYL156:PDS transformed plants turn white or not, and the no-load plants of pYL156 were injected as blank control. At about 2 weeks after VIGS infection, the leaves of pYL156:PDS plants became albino, indicating that our silencing system was stable. The expression level of *GhSNAT3D* was detected at the three-leaf stage of cotton seedlings, and it was found that the expression level of *GhSNAT3D* was significantly decreased in pYL156 as a control (Figure 8A), indicating the successful gene silencing.

PYL156 and pYL156:*GhSNAT3D* were then subjected to salt stress using 100 mM/L NaCl solution, with pYL156 as control (CK) and pYL156:*GhSNAT3D* as V-S. After 3 days of salt treatment, the plants showed a distinct phenotype (Figure 8B). Compared with CK, V-S plants were more seriously stressed, and the true leaf wilting degree of V-S plants was significantly higher than that of control plants. Because *GhSNAT3D* is a putative melatonin synthesis gene, endogenous melatonin levels were significantly reduced in silent plants (Figure 8C). It has been reported that melatonin enhances salt tolerance of cotton mainly by affecting reactive oxygen species scavenging system and Ca^2+^ signal transduction pathway (Zhang et al., 2021a). POD activity, SOD activity and Ca^2+^ content of biochemical indexes were measured on plants CK and V-S respectively (Figure 8C). The POD activity, SOD activity and Ca^2+^ content of *GhSNAT3D* gene silenced plants were significantly down-regulated by gene silencing. We detected the relative expression of some Ca^2+^ signal transduction-related genes (Figure 8D). Compared with control plants, most Ca^2+^ signal transduction-related genes were up-regulated in *GhSNAT3D* gene silenced plants, as shown in: CAM, CAMBP25, CBL4, CBP60B, CBP60B1, CML8, CML10, CPK2, CPK5; *GhSNAT3D* gene silencing up-regulated the expression of CAL1 gene. CML7 was not induced to be differentially expressed. These results indicated that *GhSNAT3D* silenced plant activated oxygen scavenging system weakened, Ca^2+^ content decreased, Ca^2+^ signal transduction related genes were differentially expressed, and salt tolerance of cotton decreased.

### Exogenous Melatonin Alleviates Salt Sensitivity of *GhSNAT*-Silenced Plants

*GhSNAT* silenced plants decreased endogenous melatonin content and increased salt sensitivity. In order to investigate whether exogenous melatonin supplementation can improve the effect of *GhSNAT3D* gene silencing on plants, we treated part of pYL156:*GhSNAT3D* plants with exogenous 20 μM/L melatonin and the same treatment. PYL156 plants were used as control (CK), pYL156:*GhSNAT3D* plants as V-S, pYL156:*GhSNAT3D* plants + melatonin as V-S'. After 3 days of salt treatment, V-S plants were more severely stressed than V-S ′plants, with cotyledons and true leaves wilting significantly in V-S plants, while cotyledons in V-S′ plants wilted slightly and true leaves lost luster (Figure 8). We measured POD activity, SOD activity and Ca^2+^ content of biochemical indexes, and found that exogenous melatonin increased POD activity, SOD activity and Ca^2+^ content of V-S plants (Figure 8). Exogenous melatonin supplementation alleviated the effect of *GhSNAT3D* gene silencing on salt tolerance of cotton, and alleviated the salt sensitivity of *GhSNAT3D* gene silencing plants.

### GhSNAT Protein Interaction Network

**FIGURE 9:**
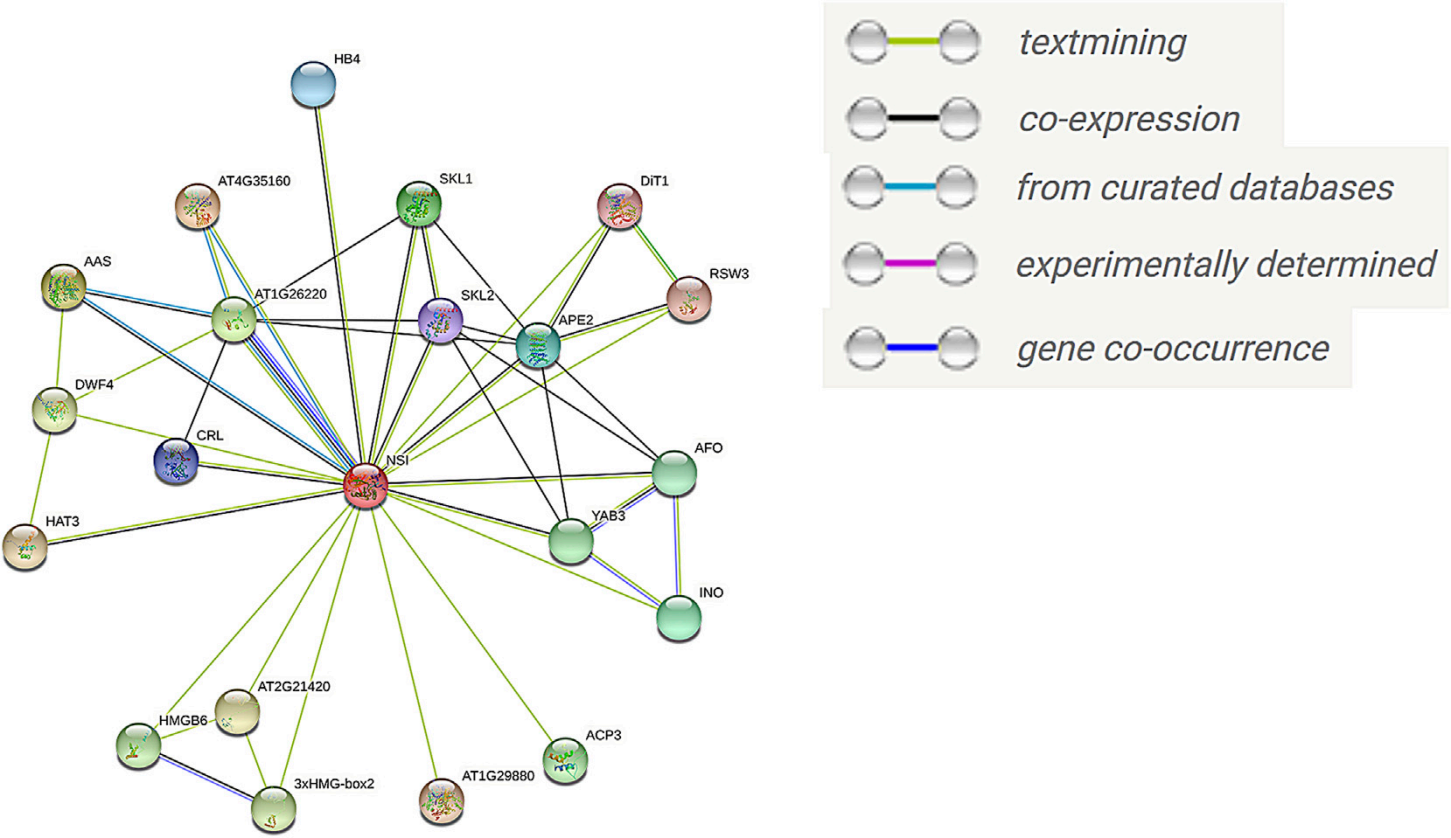
The interaction network of GhSNAT protein.

To further understand the function of GhSNAT protein, we compared GhSNAT3D protein to *Arabidopsis thaliana*, and obtained the homologous gene SNAT/NSI of *Arabidopsis thaliana* (AT1G32070.1) (Lee and Back, 2018), used the online tool STRING to predict the interaction protein of SNAT protein (Figure 9). Among these interacting proteins, AT4G35160, AAS (AT2G20340) and AT1G26220 have been identified in curated Databases to interact with *AtSNAT*. 19 proteins were predicted to interact with *AtSNAT* by textmining. Seven proteins may interact with *AtSNAT* through co-expression analysis. There was a close relationship between AT1G26220 protein and *AtSNAT* in the following aspects: co-expression, curated databases, textmining and cooccurence. Interestingly, the protein AT1G26220 has been identified as AtSNAT2 in *Arabidopsis thaliana* (Lee et al., 2019), a member of the SANT family, and its homologous gene in land cotton is GhSNAT25D. Based on these results, we speculated that GhSNAT3D and GhSNAT25D regulated melatonin levels in cotton through their interactions. Among these interacting proteins, the proteins in the Melatonin BioSynthetic Process (GO:0030187) were AT1G26220 and AT4G35160. AT4G35160 has been identified as AtASMT, the last gene for melatonin synthesis in *Arabidopsis thaliana* (Byeon et al., 2016). At the same time, the interacting proteins AT1G26220, AT4G35160 and AAS were enriched in Tryptophan Metabolism (ATH00380), and the substrate for the synthesis of melatonin was Tryptophan. Therefore, GhSNAT3D regulates the level of cotton melatonin through a complex pathway, that is, GhSNAT3D interacts with GhSNAT25D, ASMT and AAS to participate in the synthesis of melatonin.

## Discussion

During the growth and development of cotton, it is often subjected to abiotic stress. As a new phytohormone, melatonin plays an important role in plant response to abiotic stress. As a key gene in the melatonin synthesis pathway, SNAT plays an indispensable role (Kang et al., 2013). We identified *SNAT* genes in *Gossypium arboretum*, *Gossypium raimondii*, *Gossypium hirsutum*, *Gossypium barbadense* and seven other plants. This study focuses on the analysis of *SNAT* in upland cotton, with the aim of understanding the evolutionary relationship of the cotton gene family, gene family expansion, selection pressure and expression to various abiotic stresses. The results of this study will provide basic theoretical reference for further research on *SNAT* gene.

In this study, we identified family members of *SNAT* in *Gossypium arboretum*, *Gossypium raimondii*, *Gossypium hirsutum*, *Gossypium barbadense*, *Theobroma cacao*, *Arabidopsis thaliana*, *Oryza sativa*, *Populus trichocarpa*, *Vitis vinifera*, *Glycine max*, *Zea mays*, and constructed phylogenetic evolutionary trees based on their evolutionary relationships. The evolutionary tree divided these genes into five branches, and each branch haed *SNAT* gene distribution. The distribution of *SNAT* gene members indicated that *SNAT* genes were present in the ancestral genomes of both dicotyledons and monocotyledons. This also illustrates that the *SNAT* gene family had formed clade I, II, III, IV and V before the separation of dicots and monocots. Among them, Clade I contained the largest number of *SNAT*s in almost all plants, indicating that clade I may be an ancient group of *SNAT* genes, indicating its importance and contribution in the massive expansion of higher plants (Malik et al., 2020). We observed large differences in the distribution of *SNAT* among all selected plant species, for example, 33 genes in Arabidopsis, 31 genes in rice but 114 genes in soybean and 102 genes in Populus trichocarpa, suggesting that *SNAT* genes have been conserved during evolution and have undergone massive expansion in higher plants. Notably, in plants such as Arabidopsis, rice and cocoa, four cotton species have their *SNAT* proteins homologous in clades I-V, indicating that the SNAT proteins in these plants are evolutionary closely related. The SNAT proteins of tetraploid cotton (*Gossypium hirsutum* and *Gossypium barbadense*) and diploid cotton (*Gossypium arboretum* and *Gossypium raimondii*) clustered together, confirming that *Gossypium hirsutum* and *Gossypium barbadense* are the result of a cross between *Gossypium arboretum* and *Gossypium raimondii*. Phylogenetic analysis revealed that genes homologous to Rice *SNAT1* and *SNAT2* were distributed in clade II (Kang et al., 2013; Byeon et al., 2016). Subcellular localization prediction revealed that 7 *GhSNAT*s were located in chloroplasts in clades II, including *GhSNAT1A*, *GhSNAT1D*, *GhSNAT3A*, *GhSNAT6A*, *GhSNAT21A*, *GhSNAT3D* and *GhSNAT7D,* this result also suggested that melatonin synthesis occurs in chloroplasts (Tan and Reiter, 2020). It has been reported that *SNAT* is localized in chloroplasts, which is similar to what we predicted (Byeon et al., 2013).

Motif is a short sequence of relatively conserved features shared among a group of genes. It may be a recognition sequence or it may encode a functional protein (Morello and Breviario, 2008). Motif prediction can provide a basis for us to analyze the functional and structural classification of family members. The results of phylogenetic tree and motif analysis showed that most *GhSNAT* in the same branch had similar motif distribution, which provided further support for their clustering in the phylogenetic tree. In motif analysis results, all GhSNAT proteins contained motif1, which represented the conserved domain of the SNAT family, motif1 was conserved in all GhSNAT proteins. Almost all GhSNAT proteins in each clade are composed of motifs arranged in a similar way, indicating that the protein structure is highly conserved in a particular clade. The unique motifs of *GhSNAT* genes in different subfamilies may represent the conserved nature and specific functions of the GhSNAT gene family. At the same time, there were significant differences in motif composition patterns in different branches, which may be responsible for the functional specificity of SNAT proteins in different categories. Interestingly, through motif analysis of *SNAT1* and *SNAT2* in rice, we found that motif1 was the only motif composition in both of them (Kang et al., 2013; Byeon et al., 2016). Moreover, *GhSNAT3A* and *GhSNAT3D*, which are most closely related to their evolution, are only composed of motif1. We speculated that SNAT members in clade II may have the function of Serotonin n-acetyltransferase, which needs further verification. Intron is a non-coding sequence excised during the processing of precursor mRNA to mature mRNA, which accounts for a high proportion of eukaryotic genes. Introns contain not only a variety of non-coding RNA but also many regulatory elements related to gene expression (Ying et al., 2010). In the gene structure analysis, eight genes had no introns, and such genes with fewer introns can quickly evolve through replication or reverse transcription and then be integrated into the genome (Lurin et al., 2004), Genes with fewer introns are common in higher eukaryotes (Louhichi et al., 2011). Gene families with fewer introns are thought to have acquired new functions in the process of evolution through replication or reverse transcription after merging into the genome (Lurin et al., 2004). Closely related genes have similar gene structure, which may be the result of a series of gene replication (Guo et al., 2014). In this study, differences in *cis*-acting elements of genes directly affect their expression and differentiation, and the transcription factors responding to *cis*-acting elements are used to regulate gene expression. The prediction results of *cis*-acting elements showed that most of the *cis*-acting elements of *GhSNAT*s gene in upland cotton were related to environmental stress and hormone response. Most of *GhSNAT*s responded to environmental stress, which provided a reference for screening stress resistance genes. Melatonin, as a hormone, is a number of phytohormone related regulators that coordinate other phytohormone responses to salt stress in regulating the cotton defense network (Zhang et al., 2021a). More than half of *GhSNAT* members have abscisic acid responsiveness, salicylic acid responsiveness and MeJA-responsive elements. ABA, SA and MeJA may be important signals regulating GhSNAT family. It offered a possibility for melatonin to act in coordination with other phytohormones.

The chromosomal localization of *SNAT*s can clearly display the physical location distribution of each *SNAT* gene in its genome and the evolutionary relationship of some genes. There were significant differences in the number and distribution of *SNAT* in A/D genomes of *Gossypium arboreum* and *Gossypium raimondii*, which may be due to the different origins of the two cotton species. *Gossypium arboreum* is the oldest cultivated cotton variety originating from the Asian continent. *Gossypium raimondii* is a wild cotton variety grown in Peru, South America. About 1–2 million years ago, cotton with A genome similar to *Gossypium arboreum* and *Gossypium raimondii* crossed with D genome, resulting in chromosome doubling and eventually forming new At and Dt subgenome allotetraploid cotton (Wendel and Cronn, 2003). The same tetraploid *Gossypium hirsutum* has at and Dt subgenomes similar to *Gossypium barbadense*. It was shown that the number of *SNAT*s in *Gossypium hirsutum* and *Gossypium barbadense* is about twice that of *Gossypium arboretum* and *Gossypium raimondii*. Within the genome, gene replication events can be divided into two categories: tandem replication and fragment replication. The distribution of two or more genes on the same chromosome is defined as tandem duplication, while the distribution of these genes on different chromosomes is considered fragment replication (Liu et al., 2010). There was one tandem duplication on chromosome 3 of *Gossypium arboretum*, five tandem duplications on chromosome A04 of *Gossypium barbadense*, and one tandem duplication on chromosome D02 of *Gossypium barbadense*, while there was no tandem duplication in *Gossypium raimondii* and *Gossypium hirsutum*, suggesting that there was a special evolutionary pattern in the evolution of different cotton species. No tandem duplication may be the main reason why the number of *GhSNAT*s is less than *GbSNAT*s and the number of *GrSNAT*s is less than *GaSNAT*s. In this study, we found only seven very important tandem repeat gene pairs that may contribute to the inclusion of multiple introns in the repeat gene. It has previously been reported that tandem replication may involve the evolution of new genes other than introns (Iwamoto et al., 1998). In colinear analysis, 123 pairs of duplicated genes showed fragment replication and 478 pairs of duplicated genes showed genome-wide replication. The number of fragment replication in each cotton genome was much higher than that in tandem replication, indicating that fragment replication was the main driving force of *SNAT* gene amplification during evolution (He et al., 2016). As tetraploid cotton, *Gossypium hirsutum* and *Gossypium barbadense* doubled the size of the SNAT gene family through fragment and whole genome replication (WGD). Repeated gene pairs may undergo various functional differentiation throughout evolution, which may lead to the acquisition of unique traits, function partially different from the original traits and loss of the original traits (Prince and Pickett, 2002). To investigate the effect of Darwinian forward selection on post-replication differentiation of SNAT genes, and the nature and degree of selection pressure on cloned genes, we calculated Ka/Ks values of 414 gene pairs, among which Ka/Ks < 1 of 401 duplicate gene pairs was purified selection. Positive selection of 13 duplicate gene pairs with Ka/Ks > 1.96.86% of the Ka/Ks values were less than 1, so we speculated that the cotton SNAT gene family underwent strong purification selection after fragment replication, tandem replication and whole genome replication, but the functional differences were limited.

Under abiotic stress, stress response genes are induced to adapt to various developmental and physiological changes (Karanja et al., 2017). By analyzing the expression patterns of *GhSNAT*s under various stresses, we found that more than half of *GhSNAT*s were induced and responded to stress expression. There were also a few *GhSNAT*s without significant differential expression under various abiotic stresses, and their main functions may be concentrated in other aspects or some functions may be lost during evolution. There were similar expression patterns among gene pairs, and duplicate gene pairs may play an important role in adapting to the external environment and maintaining the stability of the genetic system when stimulated by the environment during evolution (Gu, 2003; Chapman et al., 2006). We analyzed the expression of *GhSNAT*s gene in different tissues. The results showed that there were differences in the expression levels of these genes. Most of the genes were tissue specific, and *GhSNAT5D*, *GhSNAT11D*, *GhSNAT13A*, *GhSNAT2D* and other genes were highly expressed in leaf. *GhSNAT1D* and *GhSNAT2A* were highly expressed in stem and petal. *GhSNAT9D*, *GhSNAT13D* and *GhSNAT12A* were highly expressed in torus. Genes that are highly expressed in specific tissues may play a role in plant growth and development (Ramamoorthy et al., 2008). *GhSNAT19A* and *GhSNAT21D* were highly expressed in each tissue, and these highly expressed genes or tissue-specific genes may play an important regulatory role in cotton development (Gu et al., 2018). Meanwhile, we detected the relative expression levels of *GhSNAT*s induced by exogenous melatonin in II. And some genes (*GhSNAT1A*, *GhSNAT3D*, *GhSNAT6A*, *GhSNAT7A*, *GhSNAT7D*, etc.) were down-regulated by exogenous melatonin. These results suggested that these genes may be related to the balance of melatonin content in plants, melatonin levels were regulated by multiple genes. Under salt stress, *GhSNAT1A* and *GhSNAT3D* were down-regulated by melatonin. We speculated that these two genes might play important roles in response to stress and regulation of endogenous melatonin. *GhSNAT7A*, *GhSNAT7D*, *GhSNAT10D*, *GhSNAT23D*, and *GhSNAT25D* were up-regulated by melatonin, suggesting that *GhSNAT*s have a complex pathway to regulate melatonin response to stress under salt stress in cotton. Through the prediction of protein network interaction, two melatonin synthesis related genes *AtSNAT2* and *AtASMT* were found in the proteins interacting with *SNAT*, and these two genes also play an important role in regulating melatonin synthesis (Byeon et al., 2016; Lee et al., 2019). Melatonin is produced from tryptophan through a continuous enzymatic reaction (Kanwar et al., 2018), and three genes of the protein interacting with *SNAT* are involved in Tryptophan metabolism. AT4G35160 and AT1G26220 have been confirmed to be involved in the regulation of the synthesis of melatonin, but there are no reports about melatonin in AAS (AT2G20340). Whether it is involved in melatonin synthesis needs further verification. Cotton regulated melatonin levels through a complex pathway, which was regulated not only by synthetic genes but also by downstream metabolic genes (Zhang et al., 2021b). In this study, *GhSNAT3D* may interact with *GhSNAT25D*, *ASMT* and AAS to regulate melatonin synthesis.

**FIGURE 10:**
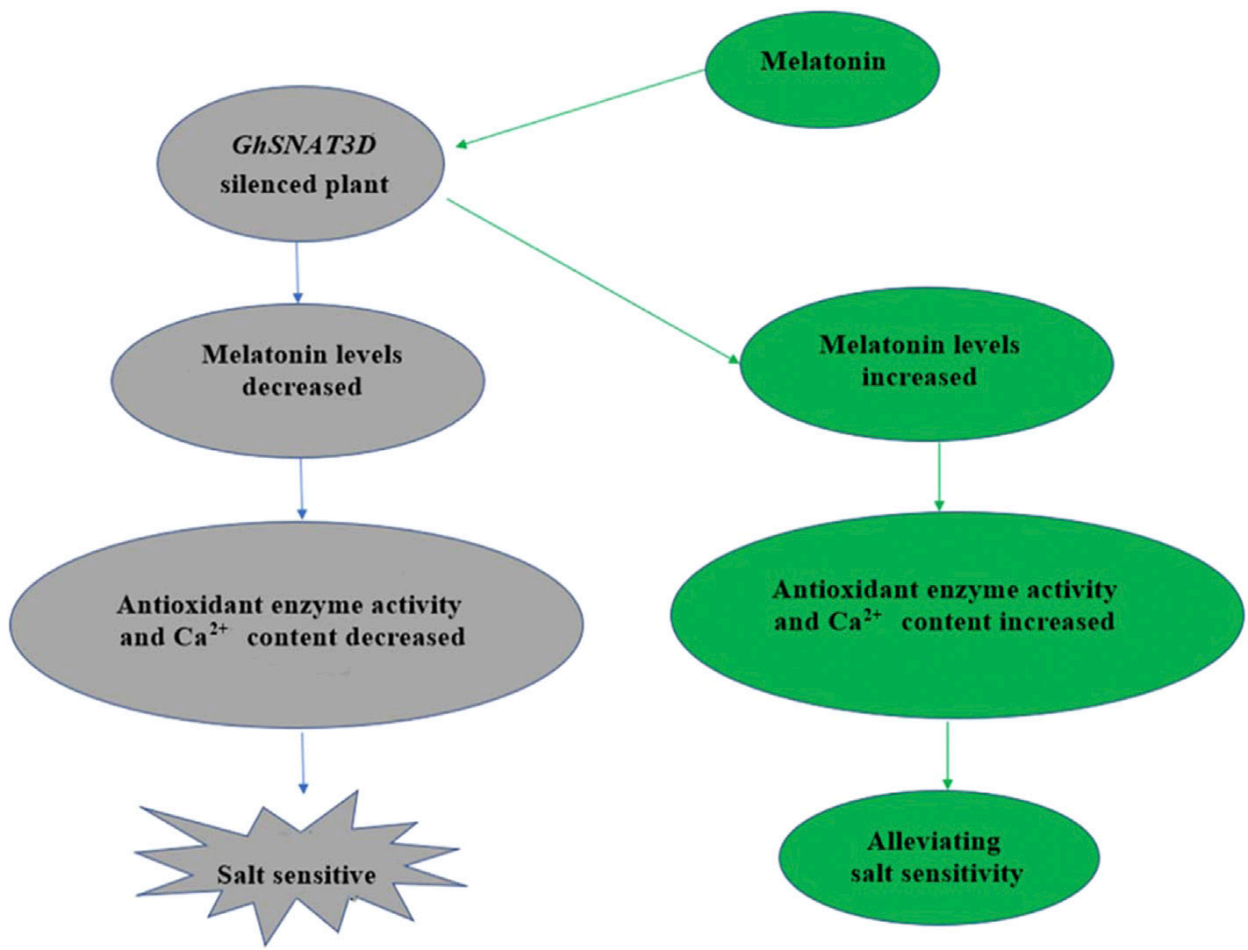
Mechanism of *GhSNAT3D* in regulating cotton response to salt stress.

Based on our results, we speculated that *GhSNAT1A* and *GhSNAT3D* might play an important role in responding to stress and regulating endogenous melatonin (Figure 10). Through further comparison with rice *SNAT* sequences, we finally selected *GhSNAT3D* as a presumed GhSNAT gene in cotton for functional verification (Kang et al., 2013). *GhSNAT3D* was silenced by virus-induced gene silencing technique, and melatonin content of silenced plants decreased significantly, suggesting that *GhSNAT3D* played an important role in regulating cotton melatonin synthesis. Under salt stress, the silent plants of *GhSNAT3D* were more seriously stressed than the control, which indicated that *GhSNAT3D* played an important role in cotton resistance to salt stress on the one hand, and that melatonin was closely related to salt tolerance of cotton on the other hand (Zhang et al., 2021a). In plants, melatonin is considered an antioxidant and plays an important role in controlling reactive oxygen species (ROS and RNS), as well as other free radicals and harmful oxidizing molecules in plant cells (Reiter et al., 2014). Melatonin may alleviate oxidative damage induced by salt stress by directly enhancing antioxidant enzyme activity or scavenging H_2_O_2_ (Li et al., 2012). The POD enzyme activity and SOD enzyme activity of *GhSNAT3D* silenced plants were significantly decreased, which was speculated to be caused by the decrease of melatonin level. Melatonin-calmodulin interactions have been shown to modulate many calcium-dependent cellular functions in animal cells (Posmyk et al., 2009), The results showed that the Ca^2+^ content of *GhSNAT3D* silenced plants decreased. Interestingly, some genes downstream of Ca^2+^ signal transduction were upregulated, which may indicate that the effect of stress on these genes was much greater than the effect of melatonin reduction. In response to external stimuli, plants increase intracellular Ca2+ concentration, which in turn activates a range of calcium-binding proteins, including Ca2^+^ sensors/decoders, protein kinases and transcription factors (Srivastava et al., 2013). The level of melatonin is directly related to the stress tolerance of plants. In addition to the synthesis *in vivo*, plants can also absorb the exogenous melatonin from the environment and accumulate it in their organs (Tan et al., 2007). Therefore, we provided exogenous melatonin to *GhSNAT3D*-silenced plants, and the results showed that the supplementation of exogenous melatonin relieved the negative effects of endogenous melatonin reduction on cotton, the POD enzyme activity, SOD enzyme activity and Ca^2+^ content of *GhSNAT3D* silenced plants were increased, so that they restored their resistance to salt stress under salt stress. *GhSNAT3D* resisted salt stress by regulating the level of endogenous melatonin in cotton.

## Conclusion

In this study, SNATs were comprehensively identified for the first time in the upland cotton genome, and 52 *GhSNAT* genes were identified, which underwent both fragment and whole genome replication during evolution. *GhSNAT*s were divided into five branches based on phylogenetic tree, gene structure and motif composition. The *GhSNAT* members in clade II may have functions of Serotonin n-acetyltransferase. It was found that in addition to tissue specificity, some *GhSNAT*s showed diversity and similarity in response to stress, and exogenous melatonin could induce differential expression of some *GhSNAT*s. By inhibiting the expression of *GhSNAT3D*, the content of melatonin in silenced plants was decreased and the salt tolerance was weakened. Exogenous melatonin supplementation alleviated the negative effects of *GhSNAT3D* gene silencing. This study provides a reference for further exploring the function of GhSNAT gene and melatonin.

## Data Availability

The original contributions presented in the study are included in the article/Supplementary Material, further inquiries can be directed to the corresponding author.
